# HPIDB 2.0: a curated database for host–pathogen interactions

**DOI:** 10.1093/database/baw103

**Published:** 2016-07-02

**Authors:** Mais G. Ammari, Cathy R. Gresham, Fiona M. McCarthy, Bindu Nanduri

**Affiliations:** ^1^School of Animal and Comparative Biomedical Sciences, University of Arizona, Tucson, AZ 85721, USA; ^2^Institute for Genomics, Biocomputing and Biotechnology, College of Veterinary Medicine, Institute for Genomics, Mississippi State University, Mississippi State, MS 39762, USA; ^3^College of Veterinary Medicine, Mississippi State University, Mississippi State, MS 39762 USA

## Abstract

Identification and analysis of host–pathogen interactions (HPI) is essential to study infectious diseases. However, HPI data are sparse in existing molecular interaction databases, especially for agricultural host–pathogen systems. Therefore, resources that annotate, predict and display the HPI that underpin infectious diseases are critical for developing novel intervention strategies. HPIDB 2.0 (http://www.agbase.msstate.edu/hpi/main.html) is a resource for HPI data, and contains 45, 238 manually curated entries in the current release. Since the first description of the database in 2010, multiple enhancements to HPIDB data and interface services were made that are described here. Notably, HPIDB 2.0 now provides targeted biocuration of molecular interaction data. As a member of the International Molecular Exchange consortium, annotations provided by HPIDB 2.0 curators meet community standards to provide detailed contextual experimental information and facilitate data sharing. Moreover, HPIDB 2.0 provides access to rapidly available community annotations that capture minimum molecular interaction information to address immediate researcher needs for HPI network analysis. In addition to curation, HPIDB 2.0 integrates HPI from existing external sources and contains tools to infer additional HPI where annotated data are scarce. Compared to other interaction databases, our data collection approach ensures HPIDB 2.0 users access the most comprehensive HPI data from a wide range of pathogens and their hosts (594 pathogen and 70 host species, as of February 2016). Improvements also include enhanced search capacity, addition of Gene Ontology functional information, and implementation of network visualization. The changes made to HPIDB 2.0 content and interface ensure that users, especially agricultural researchers, are able to easily access and analyse high quality, comprehensive HPI data. All HPIDB 2.0 data are updated regularly, are publically available for direct download, and are disseminated to other molecular interaction resources.

**Database URL:** http://www.agbase.msstate.edu/hpi/main.html

## Background

Understanding the interplay between host and pathogen that underpins health/disease enables researchers to identify potential targets for therapeutic, prophylactic and intervention strategies to eliminate or reduce the severity and economic impact of infectious diseases. Numerous biomedical studies have used network modeling of available host–pathogen interactions (HPI) to understand how pathogens manipulate host machinery and regulate cellular processes (1–4). In contrast, the number of HPI network analysis studies for agricultural pathogens in literature is scarce. A recent interactome study of Porcine Reproductive and Respiratory Syndrome Virus (PRRSV) NSP2, the largest replication protein in PRRSV, with its host cellular proteins enabled gaining functional insights into the roles of NSP2 in the replication and pathogenesis of PRRSV and identified novel cellular target proteins regulating the virus replication (5).

While there are multiple resources that report molecular interactions, identifying HPIs for important agricultural pathogens is more difficult. Commonly used databases such as DIP (6), IntAct (7) and MINT (7) for curated molecular interaction data are limited in their coverage of inter-species interactions. Some other databases such as HCVpro (8), Proteopathogen (9) for *Candida* *albicans*, and NCBI HIV-1 Human Interaction Database (10) focus on HPI but only include data on a specific pathogen. Other resources are based on a wider range of pathogens, including VirHostNet (11) for viruses, PATRIC (12) for bacteria and PHISTO (13) for human pathogens. A related resource, PHI-base (14), catalogs experimentally verified pathogenicity, virulence and effector genes from fungal, protist and bacterial pathogens that infect animal, plant and/or fungal hosts. Curated information in PHI-base indicates whether a gene affects virulence, i.e. the final outcome of a pathogen–host interaction but the database content does not have specific protein–protein interactions that could explain the molecular mechanisms of virulence.

The poor representation of HPI in molecular interaction databases is especially observed for agriculturally important pathogens, where interactions are mainly predicted based on sequence analyses without support from manual curation and assessment of interactions. Absence of species-specific HPI data impedes progress in infectious disease research, especially livestock research, toward the identification of specific targets for therapeutic strategies. Therefore, it is critical to develop resources utilizing complementary approaches of: (i) high quality, experimentally derived HPI (which also serve as the gold standard test set for computational HPI prediction) combined with (ii) rapid and accurate HPI prediction to support network modeling in a broad range of host–pathogen systems.

To provide researchers with HPI information, the Host Pathogen Interaction Database (HPIDB) was developed in 2010 (15). In this previous version, HPI data from molecular interaction databases were combined in HPIDB in a user-friendly web accessible format for querying the database content. In addition, the HPIDB homologous HPI transfer feature allowed for a first pass, rapid transfer of HPI to species that lack HPI information to support hypothesis driven research. Usage statistics indicate that the number and amount of data analysis by HPIDB users are increasing annually. During the past few years, HPIDB has been cited in multiple publications, including studies focusing on interactome and prediction of HPIs (16–19).

Since our initial development of the database, we have implemented several changes in HPIDB described in this report. To provide a unified query interface for HPI information, HPIDB 2.0 includes both data and interface updates. Modifications include biocuration of HPI from literature compliant with community standards that can serve as gold standard for HPI prediction algorithms. Our prioritization list for biocuration represents a variety of host and pathogen systems, especially pathogens capable of causing disease in agricultural species. HPIDB 2.0 results include functional information of interacting proteins and can be visualized as a network. These modified HPIDB 2.0 services ensure that researchers can easily access and visualize high quality, comprehensive HPI data.

## Providing comprehensive HPI data

HPIDB 2.0 generates a comprehensive set of HPI by (i) in-house manual curation of published, experimental HPI data and (ii) bringing in external HPI data provided by existing molecular interaction resources. Our manually curated HPI data meets community standards for data curation and is complemented by compilation of HPI data from 12 external databases to fulfill the need for a broad set of experimentally derived HPI data. Manually curated HPI can be used directly for network analysis and to improve accuracy of HPI computational prediction. At present, HPIDB 2.0 contains 45, 238 protein–protein interactions (as of February 2016) which reflects an increase in the number of host species from 49 to 70 and pathogen species from 319 to 594 compared to previous version (15). All of HPIDB 2.0 data are made publicly accessible directly and through other molecular interaction resources.

### HPI data biocuration implementation

The cumulative literature describing detailed, experimental identification of many HPI are not easily accessible for computational analysis such as network analysis and HPI prediction until it is entered into interaction databases by biocurators. To provide users with such information, we annotate experimentally derived HPI data by collecting minimum information required for network modeling and later adding the detailed experimental information that supports a full assessment of the biological context of the interactions. Host–pathogen systems targeted for curation comprise a variety of hosts and pathogens to facilitate transfer of manual curation data to other host–pathogen systems using computational methods. HPI biocuration prioritization is based upon importance of pathogens to livestock health, existing HPI data, available HPI literature and community requests. As an example, the top animal pathogens biocurated by HPIDB in 2015 are shown in Table 1 including the number of unique interactions annotated by HPIDB biocurator compared to other databases (numbers correspond to February 2016 HPIDB update).While continually annotating HPI, HPIDB biocurators also curate host–host and pathogen–pathogen interactions from literature to ensure that the publications reviewed are comprehensively annotated. Our prioritization list changes over time to keep it current with the type of HPI data required by researchers. Therefore, we encourage the scientific community to suggest relevant articles for curation by HPIDB 2.0 and work closely with HPI curators from other resources to ensure that our efforts are not duplicated.

**Table 1.** Prioritized animal pathogens for HPIDB biocuration in 2015

Pathogen	Pathogen type	Animal host(s)	Manual HPI*
Gallid herpesvirus	DNA virus	Chicken	31 (31)
Bovine herpesvirus	DNA virus	Cattle	36 (39)
Suid herpesvirus	DNA virus	Swine	179** (183)
Equine herpesvirus	DNA virus	Horse	31** (33)
*Salmonella* spp.	Gram-negative bacteria	Cattle & poultry	61 (78)

* Number of manual HPI annotations for animal pathogens annotated by HPIDB 2.0 as of February 2016. Total number of interactions from all databases is shown in brackets. Annotations may include human–host interactions.

** Interactions are from HPIDB 2.0 Community annotations.

#### Detailed, high-quality HPI curation

Our initial pathogen prioritization list is used to develop a core set of high quality detailed HPI annotations utilizing the European Bioinformatics Institute IntAct database online curation interface (7). The IntAct interface and documentation system supports manual molecular interaction annotation from literature applying a set of controlled vocabulary to generate consistent annotations that meet community standards for data sharing. HPIDB 2.0 curated HPI data exceeds the minimum standards (20), where experimental detail is available from the literature, and is consistent with the International Molecular Exchange (IMEx) consortium standards (21), a system that requires a ‘deep’ curation model. All biology is context dependent and IntAct allows biocurators to record that, e.g. a HPI only occurs for a given condition. The detailed IMEx-level curation captures experimental details for each interacting protein including their detection method (e.g. yeast two-hybrid and co-immunoprecipitation), publication, interaction type (e.g. physical interaction, association and co-localization) and interacting protein binding sites, tags, mutations, experimental and biological roles. For example, the specific interaction binding domains/motif and mutated amino acids (if available in the literature) recorded by IMEx biocurators allows the researcher to identify regions to target for disrupting a specific interaction. Furthermore, information about domain interactions provides additional information for interaction prediction. For quality control purposes, each annotation submitted to IntAct is checked by a second trained biocurator and is then assigned a confidence value based on an IntAct scoring system (22).

As of February 2016, HPIDB 2.0 added over 2,610 molecular interactions from literature, of which 71% are HPIs while the remaining are incidental intra-species interactions described in the same literature. These curated HPI annotations include HPI for multiple pathogens such as Bovine Viral Diarrhea Virus, Herpesviruses, Hepatitis C virus, *Salmonella*, among others. Due to our manual interaction biocuration effort, HPIDB 2.0 is now a contributing member of the IMEx consortium and HPIDB 2.0 interaction data is now available as part of the Proteomics Standard Initiative Common QUery Interface (PSICQUIC) (23), a web service that provides standardized access to molecular interaction databases programmatically, allowing other resources to rapidly access and disseminate this data (http://www.ebi.ac.uk/Tools/webservices/psicquic/view/main.xhtml).

Understanding the biology of infectious diseases depends on both the identification of interacting proteins, as well as on the roles that the interacting proteins play in perturbing cellular functions. However, biological functions of many host and pathogen proteins remain largely unknown. Therefore, in addition to curating molecular interaction data, HPIDB 2.0 biocurators collect Gene Ontology (GO) (24) functional information from publications to ensure comprehensive annotation of HPI and the function of the interacting proteins. This integrated approach to biocuration ensures that users have access to the detailed experimental information from HPI peer-reviewed publications.

#### Community HPI curation

The IntAct IMEx method of biocuration is labor-intensive. Therefore, to address the immediate or near-immediate needs of researchers for HPI data, we developed a community annotation data system that requires only minimum information to support network analysis. The HPIDB 2.0 system of community HPI annotation expedites network analysis and is subsequently ‘promoted’ to a detail-rich IntAct annotation by adding contextual metadata, where available.

The community HPI file contains minimum information required to visualize HPI networks and references the following data sources: accession of the pathogen protein, accession of the interacting host protein, interaction detection method and the publication reporting this interaction. We also record host and pathogen taxonomy identifiers, protein names and the molecular interaction type to facilitate searching of these files as well as standard information regarding the annotator and timeline for the annotation. As of February 2016, community annotations include Suid herpesvirus and Equine herpesvirus HPI annotations (Table 1) as well as host–host and pathogen–pathogen annotations curated from the same literature. All community annotations are added to HPIDB 2.0 data and can be queried and downloaded using ‘search by keyword’ or ‘search by sequence’ modules. Community annotations are prioritized by HPIDB biocurator for detailed IntAct-style curation. In addition, HPIDB 2.0 provides a link and contact information for researchers interested in submitting data to our community annotations, or for suggesting literature for curation. Groups submitting annotations to HPIDB 2.0 community file will be credited for their contribution.

### HPI data integration from external databases

**Figure 1.**
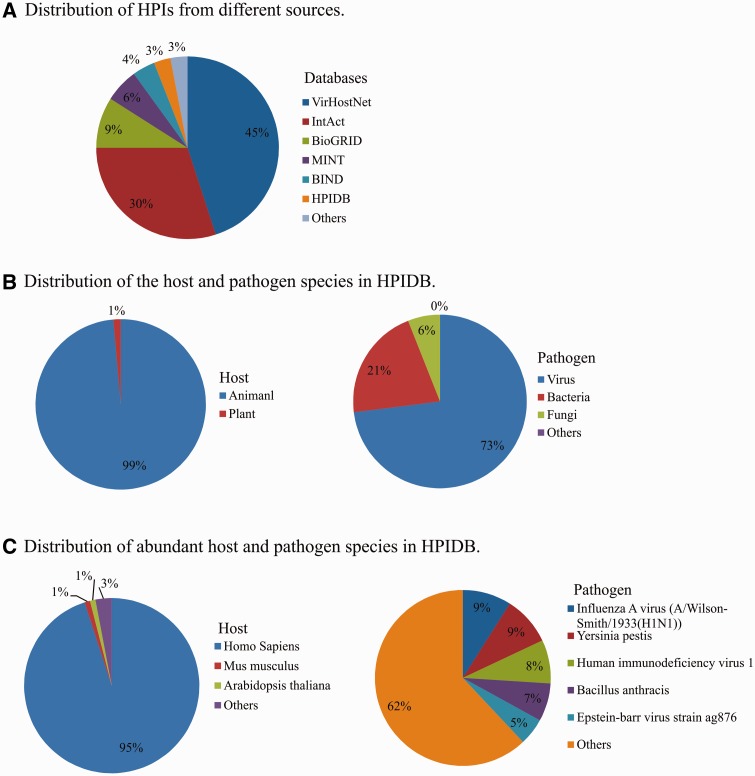
Distribution of HPIs in HPIDB 2.0 based on the source database (A), host and pathogen (B) and distribution of the species (C).

In addition to manually curated HPI, HPIDB 2.0 imports molecular interactions from other databases to generate comprehensive HPI data sets. In HPIDB 2.0 we revised our method for loading content from external interaction databases into HPIDB 2.0 to take advantage of the PSICQUIC web service, focusing only on experimentally verified interactions and giving priority to IMEx databases. This ensures that when there are multiple annotations to the same interaction pair, we preferentially select the highest quality interaction. The compiled data from other databases are mainly from IntAct (7), MINT (7), UniProtKB (25), Molecular Connections (http://www.molecularconnections.com/home/en/home/products/netPro/), MBInfo (http://www.mechanobio.info/?conversationContext=1), I2D (26), MPIDB (27), InnateDB (28), BioGRID (29), BIND (30), DIP (31), MatrixDB (32) and VirHostNet (11). Since these databases use different proteins accessions, accessions are mapped to UniProtKB or NCBI identifiers and these IDs are included in the files downloaded from HPIDB 2.0 to assist users seeking consistent interaction data sets. In addition to the accession of interacting proteins, HPIDB 2.0 also utilizes the interaction type, detection method and publication for collecting HPI from external databases. Compared to our previous strategy (15) for collecting information from relatively fewer (six databases) external sources without prioritization for loading HPI based on the quality of annotations, the current approach dramatically changes the distribution of HPIs in HPIDB 2.0 from external sources. For example, now the majority of HPIDB 2.0 data are from VirHostNet and IntAct (Figure 1A), whereas PIG (currently PATRIC) (12) and Reactome (33) were the major contributors of interactions in the initial description. All interactions data available in HPIDB 2.0, including HPIs for each host and pathogen (Figure 1B) and their species-specific data sets (Figure 1C), are freely available for download. The data growth since our previous publication (15) is attributed to the addition of new data sources in HPIDB 2.0, e.g. BioGRID, VirHostNet, HPIDB 2.0 manual annotations and an increased volume of data from external HPI sources. HPIDB 2.0 data is frequently updated in a semi-annual manner.

## User interface updates and utility

Based on user feedback, we made multiple improvements to our search and web interface services. Compared to our previous publication, the HPIDB 2.0 home page includes five modules:

- ‘Search by keyword’ allows users to search the HPIDB 2.0 database using multiple symbols/accessions,taxon, publication/author and interaction type. Improved taxon search capacity facilitates access and download of available HPI for a specific species based on taxon identifier. For each search strategy, we include sample data as examples.
- ‘Search by Sequence’ permits search with either one or multiple sequences (based upon BLAST) and allows filtering BLAST results by applying percent identity and query coverage cut-offs. The sequence search is updated with a newer version of BLAST (NCBI BLAST v2.2.31).
- ‘Search Homologous HPI’ module is used by HPIDB 2.0 to perform a sequence BLAST of users input against data in HPIDB to find homologous pairs of interacting proteins from different organisms. This enables HPIDB 2.0 users to transfer interaction annotation between species and provide a rapid, ‘first pass’ set of predicted HPI for any host and pathogen protein list. In HPIDB 2.0, the ‘Search Homologous HPI’ module is also updated with NCBI BLAST v2.2.31.
- ‘Community Annotations’ page includes a collection of HPI information generated by HPIDB 2.0 to support user request for a specific experimental data sets for network analysis as discussed earlier.
- All data in HPIDB 2.0 can be visualized in the ‘Statistics’ page in a series of dynamic charts that allow a simple ‘point and click’ download of data in a community standard MITAB2.5 file format. In addition to MITAB2.5 file, HPIDB provides users an additional file including the name, sequence and taxon identifier of interacting proteins.

In HPIDB 2.0, the two improved search modules, ‘Search by keyword’ and ‘Search by Sequence’, replace the ‘Simple search’ and ‘Advanced BLAST search’ from previous version and provide the user multiple options for a more flexible, and easy identification of specific interaction data. Each page in HPIDB 2.0 contains help information to ensure ease-of-use for a first-time visitor.

Moreover, we have expanded the HPIDB 2.0 results page to display additional information about the interaction, enabling users to assess the quality of the returned HPI. For example, HPIDB 2.0 is now able to display the information about individual mature proteins (as UniProtKB PRO identifier) from RNA viruses that produce a single polyprotein, allowing researchers to access precise information about the interacting proteins. In addition, HPIDB 2.0 results now include GO functional information for the interacting pair where available. Users can download the GO summary in a tab delimited file to use, e.g. in GOSlimViewer (34). Perhaps most noticeably for HPIDB 2.0 users is the addition of network visualization capability utilizing Cytoscape software (35) to the result page (see case study below). The HPIDB 2.0 graphical view displays protein names and allows the researcher to access additional information by hovering over nodes (proteins) and edges (interaction between two proteins) of the network. Users also have the ability to change network layout, change colors and shapes for host and pathogen proteins, filter the data (by taxon, protein identifier, detection methods, interaction types) and to export results.

### Case study: using HPIDB 2.0 for functional modeling of BVDV

**Figure 2.**
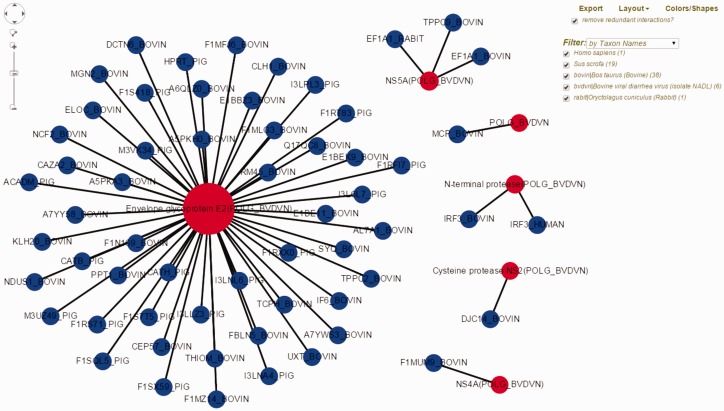
Visualization of BVDV–bovine interaction network in HPIDB 2.0. The represented network is obtained by selecting ‘Network Visualization’ in the result page obtained from a taxon search of HPIDB 2.0 data for BVDV strain NADL. The view shows the options (including exporting network, removing redundant data, changing layout, colors/shapes and filtering by taxon, protein, interaction type and detection method) available to the user to generate a custom network. Red and blue nodes represent viral and host proteins, respectively. Node size reflects the number of interactions available for proteins in the network.

Bovine Viral Diarrhea Virus (BVDV) is an important viral pathogen of cattle and infection with BVDV predisposes for secondary bacterial infections. Limited curated HPI data exists for BVDV and cattle, its host. To demonstrate the utility of HPIDB 2.0 features in studying BVDV infection, we curated over 100 HPI from available BVDV-bovine publications, of which 59 unique interactions belong to the BVDV isolate NADL. BVDV NADL data can be retrieved using ‘search by keyword’ HPIDB 2.0 module with the BVDV NADL taxon identifier (11100). All interaction information is returned as a table which can be downloaded as a MITAB file. The returned results also contain hyperlinks for each host and pathogen protein information and links to available GO information as a ‘GO summary’ file that can directly be uploaded to GOSlimViewer at AgBase (34) to summarize the available functional information. For host proteins targeted by BVDV NADL, GO terms are associated with cell death, transport, response to stress, cell differentiation and immune system process among others. Using HPIDB 2.0 ‘network visualization’ option, BVDV NADL–host interactions can be visualized in a customizable network view (Figure 2). In addition, users can find potential BVDV NADL–host interactions by using the HPIDB 2.0 ‘search by homologous’ module. Using BVDV NADL polyprotein and individual viral protein sequences as an input to BLAST (*E* value < 10 ^−^ ^6^, sequence coverage > 50%, sequence similarity > 50%) against HPIDB 2.0 data shows 167 possible BVDV–host interactions.

## Discussion and conclusions

Animal pathogens are closely linked to animal health and disease, and are zoonotic pathogens of human via environment, thus they fall under the realm of the OneHealth initiative (36). While there are an increasing number of databases that provide HPI data, most focus on specific pathogens, usually human pathogens. As shown in Figure 1, 95% of HPI data from all sources in HPIDB 2.0 are based upon human HPI, with a scarcity of curated data to support network analysis in other species, including species that are critical for agriculture. The availability of easily accessible HPI to a few species results in a critical analysis bottleneck, as outside of these few species, network analyses to identify intervention strategies is incomplete and based mainly upon transfer of interactions from model species without annotation of HPI for species of interest. Our approach of providing targeted annotation of HPI data and complementing this data with annotations from external resources provides comprehensive set of annotated HPI data. For example, there are several animal viruses with experimentally verified molecular interactions (including, animal herpesviruses) that were not found earlier in any interaction databases before our curation effort.

In addition, >80% of our HPI manual annotations are unique, emphasizing the impact of our curation approach. The significance of HPIDB 2.0 curation is also shown by the use of our annotations in recent studies [e.g. analysis HCV–host protein–protein interaction network (37)] and the integration of HPIDB 2.0 annotations into other databases [e.g. the VirHosNet (11)]. Although our curation efforts are relatively new, HPIDB 2.0 is already the sixth largest supplier of manually curated HPI data.

HPIDB 2.0 database is implemented using MYSQL; this relational database schema can easily be expanded to include additional features. While HPIDB 2.0 services ensures identifying comprehensive HPI using three different approaches (curation, data integration and homologous transfer), it also recognizes researchers need to visualize and analysis such data. HPIDB 2.0 graphical viewer provides unique opportunities for users to construct a custom host–pathogen protein interaction network and generate figures for publication in few steps. In addition, availability of GO data to the results allows the users to add functional information to identified interactions.

The HPIDB 2.0 is regularly updated (bi-annually) to ensure that researchers have continued access to the most up-to-date and comprehensive HPI data. Regular updates also allow implementation of additional functionality to HPIDB 2.0 based on user input. Moreover, disseminating our data to other molecular interaction resources ensures that researchers have multiple access points for our data.

In conclusion, the HPIDB 2.0 resource facilitates both the identification and functional analysis of HPI for a broad range of pathogens and their hosts. HPIDB 2.0 provides targeted curation, integration with existing HPI data from external sources and tool(s) to predict additional HPI where annotated data is scarce. This unique quality of HPIDB 2.0 ensures that researchers have access to the most comprehensive data set for their system and avoid the time-consuming series of steps required to integrate, standardize and annotate HPI data. As a member of the IMEx consortium, HPIDB 2.0 annotations meet community standards to provide detailed contextual experimental information and facilitate data sharing between molecular interaction resources. The data updates are accompanied with enhanced web interface that allows the users to search, visualize, analyse and download HPI data. We encourage researchers to contact us (agbase@igbb.msstate.edu) to request additional annotations or for user support. In the future, the addition and integration of yet more data types and features will further increase the efficiency of HPIDB 2.0 in identifying, analysing and predicting HPIs for all infectious diseases.

## Future directions

Our future goals for HPIDB 2.0 include broadening the number of pathogens for which experimentally derived manual curation HPI data is available and enabling the end user to evaluate the quality of transferred homologous HPI. By increasing the number of pathogens for which HPI data are available, we will (i) enable more infectious disease researchers to use our data and (ii) provide a more comprehensive ‘gold standard’ set of HPI that can serve as the basis for improving computational HPI prediction. Improving HPI prediction in turn will enable a more diverse group of researchers to rapidly generate HPI for network analysis and for hypothesis testing. We will also continue working to meet community needs for additional HPI data and to collaborate with existing HPI curation efforts to ensure quality and dissemination of our interaction data.

## Availability and requirements

HPIDB 2.0 is available at http://www.agbase.msstate.edu/hpi/main.html. The database is freely accessible and only requires the input data from the user. HPIDB 2.0 curated molecular interaction data is freely available without restriction form HPIDB 2.0 or PSICQUIC.
